# Prognosis prediction in esophageal signet-ring-cell carcinoma: a competing risk analysis

**DOI:** 10.1186/s12876-023-02818-z

**Published:** 2023-05-23

**Authors:** Chen Chen, Zehua Wang, Yanru Qin

**Affiliations:** 1 Department of Oncology, The First Affiliated Hospital of Zhengzhou University, Zhengzhou, China; 2 State Key Laboratory of Esophageal Cancer Prevention and Treatment and Henan Key, Zhengzhou University, Zhengzhou, China

**Keywords:** Esophageal carcinoma, Signet-ring-cell, Competing risk nomogram, Prognosis prediction, SEER

## Abstract

**Objective:**

This study aims to construct and validate a competing risk nomogram model to predict 1-year, 3-year, and 5-year cancer-specific survival (CSS) for patients with esophageal signet-ring-cell carcinoma.

**Methods:**

Patients diagnosed with esophageal signet-ring-cell carcinoma (ESRCC) between 2010 and 2015 were abstracted from the Surveillance, Epidemiology, and End Results (SEER) database. We performed the competing risk model to select significant variables to build a competing risk nomogram, which was used to estimate 1-year, 3-year, and 5-year CSS probability. The C-index, receiver operating characteristic (ROC) curve, calibration plot, Brier score, and decision curve analysis were performed in the internal validation.

**Results:**

A total of 564 patients with esophageal signet-ring-cell carcinoma fulfilled the eligibility criteria. The competing risk nomogram identified 4 prognostic variables, involving the gender, lung metastases, liver metastases, and receiving surgery. The C indexes of nomogram were 0.61, 0.75, and 0.70, respectively for 5-year, 3-year, and 1-year CSS prediction. The calibration plots displayed high consistency. The Brier scores and decision curve analysis respectively favored good prediction ability and clinical utility of the nomogram.

**Conclusions:**

A competing risk nomogram for esophageal signet-ring-cell carcinoma was successfully constructed and internally validated. This model is expected to predict 1-year, 3-year, and 5-year CSS, and help oncologists and pathologists in clinical decision making and health care management for esophageal signet-ring-cell carcinoma patients.

## Introduction

Esophageal cancer (EC) is a common malignancy of digestive system and is reported to have 604100 new cases globally in 2020, with Asia accounting for 79.7% [1]. Although esophageal cancer is often diagnosed as squamous-cell carcinomas, [2, 3] the incidence of adenocarcinoma has increased over the past two decades [4]. Esophageal signet-ring cell carcinoma (ESRCC) is a special subtype of esophageal adenocarcinoma characterized by the abundant accumulation of intracellular mucin, which replaces and compresses the nucleus to the periphery of the cell, thereby forming the classical signet-ring shape [5]. As reported in the western studies, the incidence of ESRCC is estimated to float from 3.5% to 5% of all esophageal malignancies [6–8].

The current understanding of prognostic implications of ESRCC is limited and somewhat inconsistent. Owing to most SRC carcinoma deriving from stomach or gastroesophageal junction, the prognosis of SRC carcinoma primarily originated from esophagus is not well studied [9]. Compared to usual-type esophageal adenocarcinoma, available literature pointed out that ESRCC patients tend to present with more aggressive feature, less responsive to induction therapy, and have worse overall survival (OS) and disease-free survival (DFS) after surgery, all of which support SRC histology as a predictive marker for poor prognosis [6, 7, 10–12].

However, one research from the United Stated claimed that SRC does not necessarily portend a worse prognosis [13]. These discrepancies motivate more efforts to provide an overview of survival analysis and find out potential risk factors affecting long-term prognosis. In addition, large population-based study never specifically spotlighted on the prognosis prediction model for ESRCC. The Surveillance, Epidemiology, and End Results (SEER) database covering around 28% U.S. population provides a relatively large sample-based database for exploring such a rare cancer type [14]. Moreover, there are frequently multiple time-to-event outcomes in clinical follow-up. Death is not only attributed to primary tumor or relapse, but also caused by non-ESRCC-specific reasons, such as toxicity of interventions patients underwent. These different outcomes are in competition and might lead to result bias if only performing Cox proportional hazard model. Therefore, the competing risk analysis is more appropriate when dealing with multiple event outcomes.

To address above issues, this study is to identify risk factors affecting cancer-specific survival (CSS) in patients with ESRCC. Besides, we aim to construct and validate a competing risk nomogram model in SEER database to predict 1-year, 3-year, and 5-year cancer-specific survival.

## Materials and methods

### Study population

**Fig. 1:**
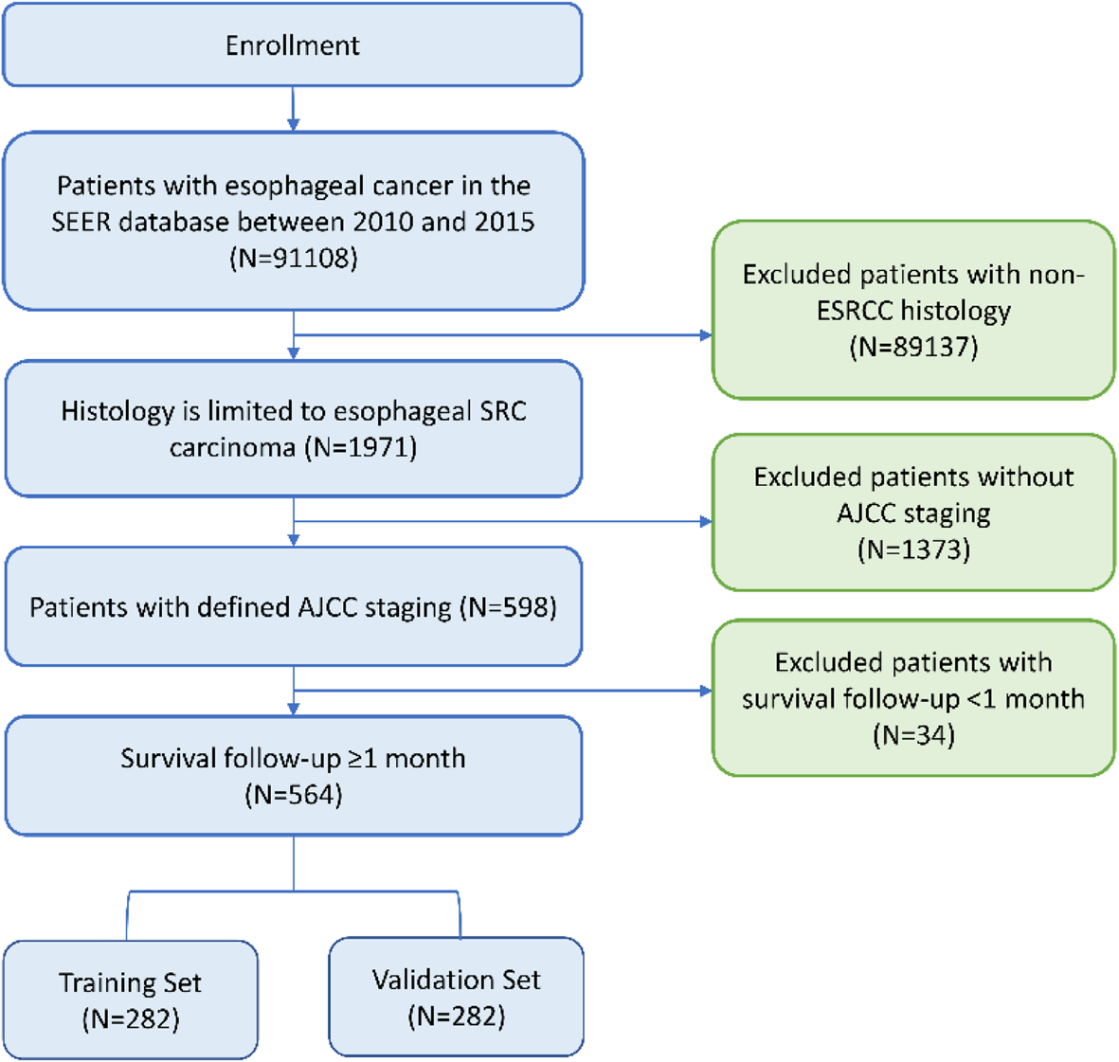
Flowchart of study design and patient selection

The Surveillance, Epidemiology, and End Results (SEER) Program is one of the largest population-based cancer databases, covering approximately 27.8% of the U.S. population [14]. Because of the de-identified nature of SEER database, local institutional ethics committee and informed consents were not required. All patient data were obtained from the SEER database using SEER*Stat, version 8.3.8. A total of 90544 patients were excluded due to missing or incomplete clinic pathological data. Eligible patients (*N* = 564) with ESRCC were randomly divided into training set (*N* = 282) and validation set (*N* = 282) with a ratio of 1:1. A detailed inclusion algorithm is shown in Fig. 1.

The inclusion criteria were set as follows: 1) Patients with esophageal cancer diagnosed between 2010 and 2015; 2) Histology ICD-O-3 (International Classification of Diseases for Oncology) limited to ESRCC (8490/3); 3) Patients with a defined clinical stage (American Joint Committee on Cancer Staging Manual, the 7^th^ edition) including stage I-IV; 4) Follow-up time ≥ 1 month.

### Predictive variates and main outcomes

The study covariates were collected and sorted: age at diagnosis (≤ 65 and > 65), sex (male and female), race (white and others), marital status (divorced, married, single, and widowed), history of malignancy (newly and previously), primary tumor site (15-32 cm, 33–40 cm, and others), grade of differentiation (I-II and III-IV), AJCC stage (I, II, III, and IV), T stage (T1, T2, T3, T4, and TX), N stage (N0, N1, N2, and N3/NX), regional lymph node examined (0, 0–10, and 11–60), positive regional lymph nodes (0, 0–2, 3–35), bone metastases (no and yes), liver metastases (no and yes), lung metastases (no and yes), surgery (no and yes), radiotherapy (no and yes), chemotherapy (no and yes).

Death owing to ESRCC was deemed as CSS. Death due to non-cancer reasons were regarded as other-cause death. Overall survival (OS) was defined as the period from the date of initial diagnosis to the date of death or the last follow-up.

### Statistical analysis

All statistical analyses were performed with R software version 4.0.5. A *P*-value of < 0.05 was considered statistically significant.

Covariates were presented as frequency (percentages) and were compared by chi-square test. Cumulative incidences of death (CID) for both ESRCC and non-ESRCC were assessed by the cumulative incidence function (CIF) model. We used the proportional sub distribution hazard (SH) model to quantify the influences of covariates on ESRCC, which is a semi-parametric model considering competing risks. We used the “cmprsk” R package to perform the competing risk analysis. Stepwise regression was conducted in multivariate competing risk analysis to find out the significant variables. The measures of prognostic value on ESRCC-related mortality were displayed with hazard ratios (HR) and 95% confidence interval (CI). All variates were selected based on the minimum Bayesian Information Criterions (BIC) principle. Finally, the determined factors were used to construct a competing risk nomogram model.

Internal validation of the nomogram model was carried out using the validation cohort. Calibration and discrimination are two important aspects to predict the accuracy of the competing risk model. First, the concordance index (C-index) was used to evaluate the prediction accuracy of the established model, ranging from 0.5 (no discrimination) to 1.0 (best discrimination). The Area Under the Curve (AUC) value was measured to assess discrimination ability at the given time. The AUC value was between 0.5 and 0.6, 0.6 and 0.7, or greater than 0.8, manifesting poor, moderate, or good prediction performance, respectively. Second, calibration curves were used to evaluate the consistency between the “predicted value” and the “actual value”. Third, we assessed the Brier score of the nomogram to make a comprehensive evaluation. Finally, we used decision curve analysis (DCA) to judge clinical utility of competing risk model. In DCA curves, threshold probability (X-axis) is the minimum probability of which further strategy would be warranted, and Y-axis represents net benefit that is calculated by subtracting harms form benefits. There are two control curves, representing all patients were treated with the competing risk nomogram and none, respectively.

## Results

### Patients characteristics

A total of 564 patients with ESRCC fulfilled the eligibility criteria between 2010 and 2015 from the SEER database and were randomly divided into the training cohort (*N* = 282) and the validation cohort (*N* = 282). The demographics and clinicopathological characteristics for two groups are presented in Table 1.

**Table 1:** Baseline characteristics of patients diagnosed with esophageal signet-ring-cell carcinoma

Variables	Total (*n* = 564)		Training (*n* = 282)		Validation (*n* = 282)		*P* value
	**No**	**%**	**No**	**%**	**No**	**%**	
**Age at Diagnosis**							0.063
Mean ± SD	66.64 ± 10.9		65.96 ± 11.09		67.31 ± 10.69		0.142
Age < = 65	261	46	142	50	119	42	
Age > 65	303	54	140	50	163	58	
**Sex**							0.523
Female	70	12	38	13	32	11	
Male	494	88	244	87	250	89	
**Race**							1
Others	41	7	20	7	21	7	
White	523	93	262	93	261	93	
**Marital Status**							0.892
Divorced/Separated	55	10	29	10	26	9	
Married	343	61	167	59	176	62	
Single/Unmarried	93	16	48	17	45	16	
Widowed/Others	73	13	38	13	35	12	
**History of Malignancy**							1
Newly	543	96	272	96	271	96	
Previously	21	4	10	4	11	4	
**Primary Site**							0.507
15-32 cm	42	7	20	7	22	8	
33-40 cm	468	83	231	82	237	84	
Others	54	10	31	11	23	8	
**Grade**							0.979
I-II	25	4	13	5	12	4	
III-IV	457	81	228	81	229	81	
Others	82	15	41	15	41	15	
**AJCC Stage**							0.26
I	67	12	26	9	41	15	
II	120	21	60	21	60	21	
III	192	34	101	36	91	32	
IV	185	33	95	34	90	32	
**Tumor Size**							0.114
T1	133	24	56	20	77	27	
T2	59	10	26	9	33	12	
T3	243	43	127	45	116	41	
T4	74	13	44	16	30	11	
TX	55	10	29	10	26	9	
**Node Status**							0.823
N0	196	35	103	37	93	33	
N1	257	46	124	44	133	47	
N2	64	11	31	11	33	12	
N3/NX	47	8	24	9	23	8	
**Regional Nodes Examined**							0.344
0	415	74	204	72	211	75	
0–10	52	9	31	11	21	7	
11–60	97	17	47	17	50	18	
**Positive Lymph Nodes**							0.682
0	485	86	240	85	245	87	
0–2	44	8	22	8	22	8	
3–35	35	6	20	7	15	5	
**Bone Metastases**							1
NO/Unknown	514	91	257	91	257	91	
YES	50	9	25	9	25	9	
**Liver Metastases**							1
NO/Unknown	516	91	258	91	258	91	
YES	48	9	24	9	24	9	
**Lung Metastases**							0.693
NO/Unknown	537	95	267	95	270	96	
YES	27	5	15	5	12	4	
**Surgery**							0.855
NO	391	69	194	69	197	70	
YES	173	31	88	31	85	30	
**Radiotherapy**							0.495
NO	424	75	208	74	216	77	
YES	140	25	74	26	66	23	
**Chemotherapy**							0.213
NO	148	26	67	24	81	29	
YES	416	74	215	76	201	71	
**Outcomes**							0.639
Survival	113	20	52	18	61	22	
Cancer-specific death	355	63	181	64	174	62	
Other-cause death	96	17	49	17	47	17	

A majority of ESRCC patients were men in both training set (87%) and validation set (89%, *P* = 0.523). Approximately 93% patients were white among selected population. More than half of the patients (61%) were married. Most patients with ESRCC were newly diagnosed (96%) versus only 4% previously confirmed. Primary tumor sites were frequently detected at 33–40 cm (83%) from the incisors. The proportion of patients who had grade III-IV (81%) ESRCC was remarkably higher than other differentiation grades. Fewer ESRCC patients presented with distant metastasis, regardless of whether the metastases located in the brain, liver, or lung. In the whole group, a lower proportion of patients received surgery (31% VS. 69%) or radiotherapy (25% VS. 75%), while a higher percentage of patients underwent chemotherapy (74% VS. 26%).

However, there are no statistically significant differences (all *p* > 0.05) between two cohorts in terms of the age at diagnosis (*P* = 0.063), sex (*P* = 0.523), race (*P* = 1.000), marital status (*P* = 0.892), history of malignancy (*P* = 1.000), primary site (*P* = 0.507), grade (*P* = 0.979), AJCC stage (*P* = 0.260), tumor size (*P* = 0.114), node status (*P* = 0.823), regional nodes examined (*P* = 0.344), positive lymph nodes (*P* = 0.682), bone metastasis (*P* = 1.000), liver metastasis (*P* = 1.000), lung metastasis (*P* = 0.693), surgery (*P* = 0.855), radiotherapy (*P* = 0.495), chemotherapy (*P* = 0.213). Therefore, the baseline characteristics were well balanced between two groups.

### Cumulative incidence of ESRCC

**Fig. 2:**
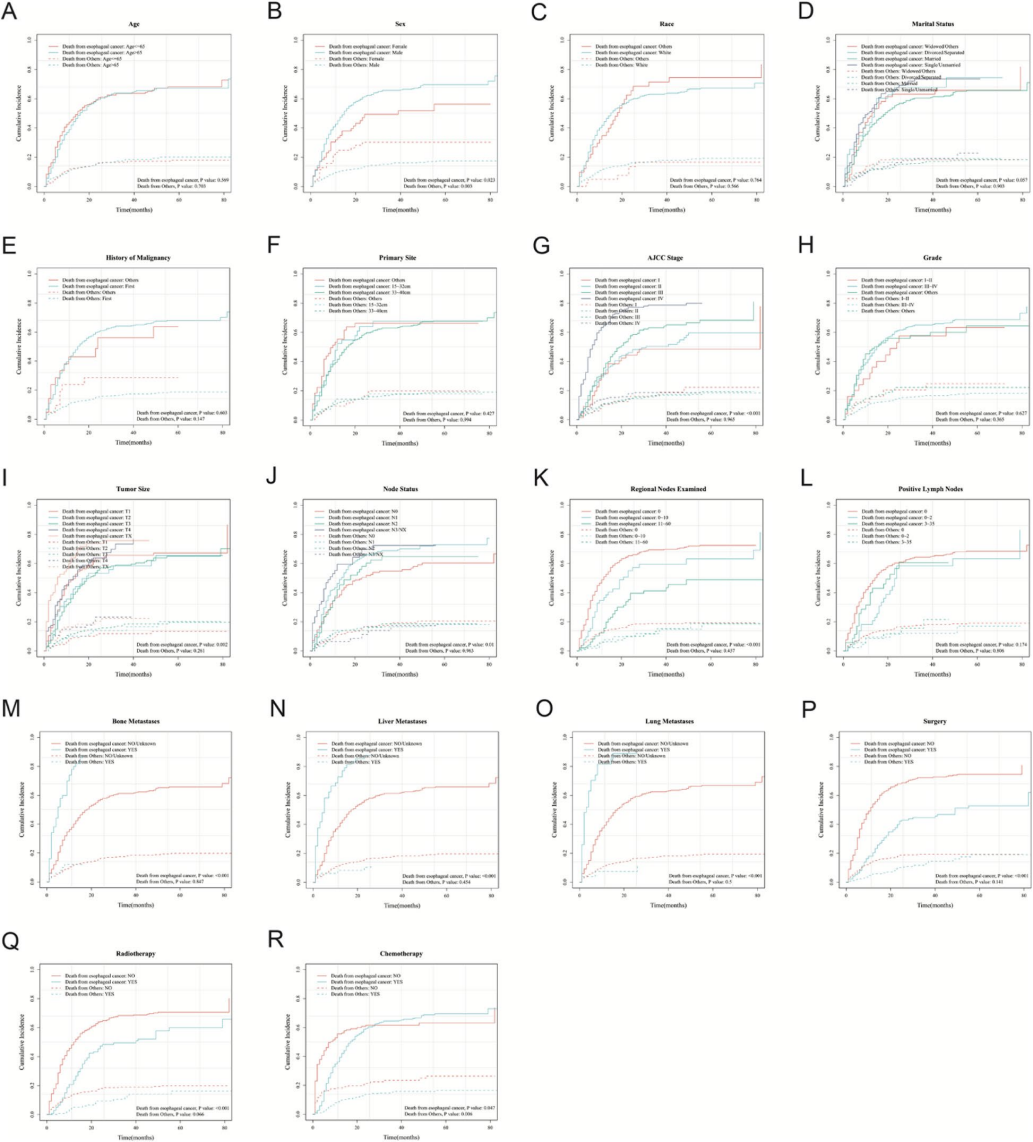
Cumulative incidence plot depicting cancer-caused death and other cause mortality based on clinicopathological features. Subgroups are respectively age (**A**), sex (**B**), race (**C**), material status (**D**), history of malignancy (**E**), primary site (**F**), AJCC stage (**G**), grade (**H**), tumor size (**I**), node status (**J**), regional nodes examined (**K**), positive lymph nodes (**L**), bone metastases (**M**), liver metastases (**N**), lung metastases (**O**), surgery (**P**), radiotherapy (**Q**), chemotherapy (**R**)

We classified the time-to-event outcomes into two endpoints: cancer-specific death and other-cause death. For all included patients (*N* = 564), there were 355 ESRCC-specific deaths, 96 other causes of deaths, and 113 alive patients during the follow-up. The 5-year cumulative incidence plots depicting cancer-caused death and other causes death are presented in Fig. 2.

When subgroup analyses were conducted based on clinicopathological characteristics, results showed that the age, race, marital status, history of malignancy, primary site, grade, positive lymph nodes had no association (*P* > 0.05) with 5-year cumulative mortality. However, for cancer-specific deaths, the 5-year cumulative curves showed statistical differences in the subgroup of sex (*P* = 0.023), AJCC stage (*P* < 0.001), tumor size (*P* = 0.002), node status (*P* = 0.01), regional nodes examined (*P* < 0.001), bone metastasis (*P* < 0.001), liver metastasis (*P* < 0.001), lung metastasis (*P* < 0.001), receiving surgery (*P* < 0.001), receiving radiotherapy (*P* < 0.001), receiving chemotherapy (*P* = 0.047). It can be inferred that above mentioned covariates were associated with cancer-specific survival (*P* < 0.05). But not all of factors had significant associations with other reasons of deaths.

### Multivariate SH model of ESRCC

To reduce the impact of competing risk bias, we performed the competing risk analysis with multivariate sub-distribution hazard function (Fine-Gray model) to quantify the effect of covariates on ESRCC (Table 2).

**Table 2:** Multivariate SH model in the training set and the validation set

Characteristics	Training Cohort (*N* = 282)			Validation Cohort (*N* = 282)		
	SHR	95% CI	*P* value	SHR	95% CI	*P* value
**Age**						
≤ 65	Reference			Reference		
> 65	0.93	0.676–1.28	0.66	1.139	0.823–1.576	0.43
**Sex**						
Female	Reference			Reference		
Male	2.309	1.259–4.236	**0.007**	1.641	0.893–3.017	0.11
**Race**						
Others	Reference			Reference		
White	0.712	0.436–1.165	0.18	1.164	0.683–1.985	0.58
**Marital status**						
Widowed/others	Reference			Reference		
Divorced/Separated	1.033	0.523–2.042	0.92	0.885	0.448–1.748	0.73
Married	0.878	0.539–1.428	0.6	0.767	0.469–1.255	0.29
Single/Unmarried	0.963	0.525–1.766	0.9	0.947	0.539–1.662	0.85
**History of Malignancy**						
Previously	Reference			Reference		
Newly	1.897	0.652–5.52	0.24	0.715	0.284–1.802	0.48
**Primary Site**						
Others	Reference			Reference		
15-32 cm	1.244	0.625–2.476	0.53	1.55	0.527–4.588	0.43
33-40 cm	0.647	0.376–1.113	0.12	2.612	1.182–5.772	**0.018**
**Grade**						
I-II	Reference			Reference		
III-IV	1.143	0.485–2.696	0.76	1.314	0.716–2.412	0.38
Others	1.252	0.5–3.134	0.63	1.184	0.592–2.37	0.63
**AJCC Stage**						
I	Reference			Reference		
II	1.564	0.678–3.609	0.29	2.069	0.977–4.38	0.058
III	1.899	0.769–4.686	0.16	4.336	1.883–9.987	**< 0.001**
IV	2.358	0.99–5.614	0.053	3.173	1.435–7.017	**0.004**
**Tumor Size**						
T1	Reference			Reference		
T2	1.117	0.599–2.085	0.73	0.676	0.337–1.355	0.27
T3	0.799	0.489–1.306	0.37	0.388	0.218–0.691	**0.001**
T4	0.819	0.439–1.529	0.53	0.441	0.235–0.827	**0.011**
TX	0.608	0.319–1.157	0.13	0.65	0.32–1.32	0.23
**Node Status**						
N0	Reference			Reference		
N1	1.103	0.685–1.775	0.69	0.71	0.444–1.136	0.15
N2	0.952	0.51–1.779	0.88	0.548	0.285–1.054	0.072
N3/NX	1.128	0.54–2.354	0.75	0.588	0.251–1.376	0.22
**Regional Nodes Examined**						
0	Reference			Reference		
0–10	1.286	0.653–2.533	0.47	0.962	0.297–3.112	0.95
11–60	1.007	0.482–2.106	0.98	0.408	0.128–1.304	0.13
**Positive Lymph Nodes**						
0	Reference			Reference		
0–2	1.006	0.523–1.936	0.99	1.92	0.809–4.588	0.14
3–35	1.537	0.706–3.346	0.28	2.949	0.94–9.253	0.064
**Bone Metastases**						
NO/Unknown	Reference			Reference		
YES	1.105	0.614–1.988	0.74	2.125	1.215–3.717	**0.008**
**Liver Metastases**						
NO/Unknown	Reference			Reference		
YES	1.573	0.848–2.919	0.15	2.238	1.301–3.85	**0.004**
**Lung Metastases**						
NO/Unknown	Reference			Reference		
YES	2.364	1.187–4.709	**0.014**	1.552	0.743–3.241	0.24
**Surgery**						
NO	Reference			Reference		
YES	0.497	0.271–0.91	**0.024**	0.331	0.116–0.948	**0.039**
**Radiotherapy**						
NO	Reference			Reference		
YES	1.098	0.616–1.956	0.75	1.991	1.014–3.906	**0.045**
**Chemotherapy**						
NO	Reference			Reference		
YES	0.82	0.498–1.351	0.44	0.757	0.491–1.168	0.21

In the training set (*N* = 282), sex (HR = 2.309, *P* = 0.007) and lung metastases (HR = 2.364, *P* = 0.0014) were independent risk factors associated with CSS, while receiving surgery (HR = 0.497, *P* = 0.024) was a protective factor correlated with better prognosis. Other covariates had no apparent associations with ESRCC (*P* > 0.05). Also, in the validation set (*N* = 282), the primary tumor located at 33–40 cm from incisor (HR = 2.612, *P* = 0.018), AJCC stage III (HR = 4.336, *P* < 0.001) and stage IV (HR = 3.173, *P* = 0.004), bone metastasis (HR = 2.125, *P* = 0.008), liver metastases (HR = 2.238, *P* = 0.004), receiving radiotherapy (HR = 1.991, *P* = 0.045) had a correlation with high risk of cancer-specific deaths, while T3 (HR = 0.388, *P* = 0.001), T4 (HR = 0.441, *P* = 0.011), and receiving surgery (HR = 0.331, *P* = 0.039) were associated with superior prognosis for ESRCC patients. The independent prognostic factors were not exactly the same from two cohorts owing to the heterogeneity of death events. But both two sets confirmed that protective surgery might be effective measures for ESRCC to achieve better prognosis.

### Construction of a nomogram model

The variable selection was performed by stepwise regression. We constructed various models with different combinations of prognostic factors. Following the minimum Bayesian Information Criterions (BIC) principle, we chose the best fitting model and the corresponding variables were included. Explicitly, sex, surgery, lung metastases, liver metastases were identified as the significant variables. Based on the estimated coefficients, surgery acted as a protective factor (coefficient = -0.654), while male (coefficient = 0.708), liver metastasis (coefficient = 0.588), and lung metastasis (coefficient = 0.942) were independent risk factors.

**Fig. 3:**
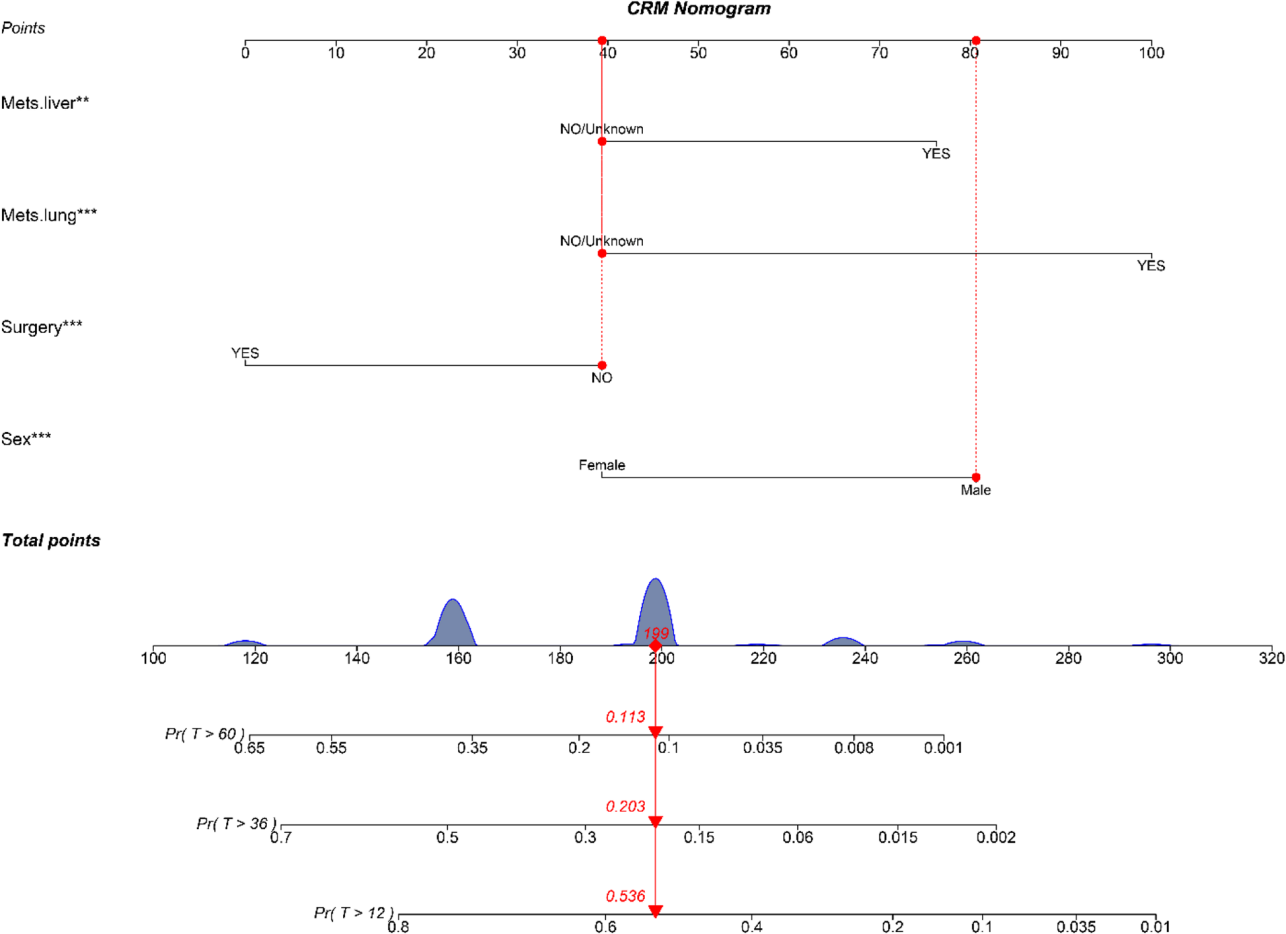
A competing risk nomogram predicting 1-year, 3-year, and 5-year cancer-specific survival probability of patients with esophageal signet-ring-cell carcinoma. ***P < 0.05, **P < 0.1

A competing risk nomogram model (Fig. 3) was built with the weighted score and the variables mentioned above. The specific value for each factor was labeled with a straight line drawn upwards to the “point axis”. The total score could be calculated by adding up the points of all variables. We could easily estimate the probability of 5-year, 3-year, and 1-year ESRCC-specific survival by locating the “total point axis” down straight to the “survival axis”.

### Validation of a nomogram model

For validation of the accuracy and stability of nomogram, we built two models for comparison. The first was a model composed of all covariates (ALL-model), and the second was a nomogram model based on BIC screening (BIC-model). We explored whether the BIC-based nomogram displayed better discrimination, calibration, and clinical benefits than the ALL-model. The nomogram was constructed with the data from the training set, while the validation set was used to validate the nomogram.

#### Discrimination and calibration

**Fig. 4:**
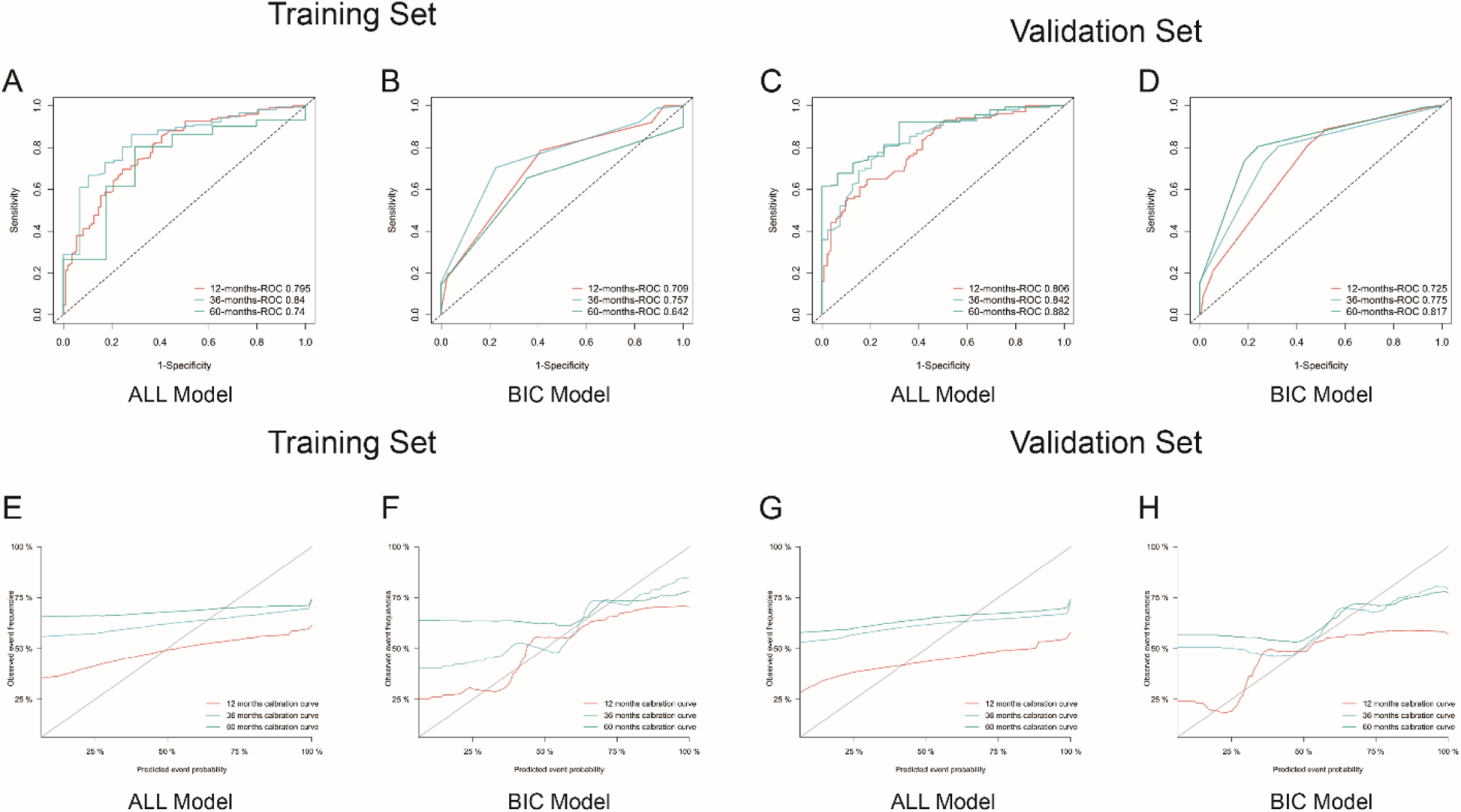
Receiver operating characteristics (ROC) curves and calibration plots for the 1-, 3-, and 5-year cancer-specific survival of BIC-nomogram and ALL-model. (**A**-**B**) ROC curves of the training set; (**C**-**D**) ROC curves of the validation set; (**E**–**F**) calibration plots of the training set; (**G**-**H**) calibration plots of the validation set. AUC, area under the curve

The nomogram model showed adequate discrimination with C-indexes of 0.61, 0.75, and 0.70 respectively for 5-year, 3-year, and 1-year CSS prediction. Receiver operating characteristic (ROC) curves were plotted in Fig. 4. The Area Under the Curve (AUC) was used to judge the accuracy of the prediction model. In the training set, the AUCs of nomogram (Fig. 4B) and All-model (Fig. 4A) were respectively 0.709 and 0.795 for 1-year, 0.757 and 0.84 for 3-year, and 0.642 and 0.74 for 5-year. Evidently, the nomogram had better prediction accuracy for 1- and 5-year survival probability (AUC > 0.7) than that of 3-year (0.5 < AUC < 0.7). However, all AUCs of nomogram were slightly smaller than the corresponding values of ALL-model. Similar outcomes could be observed in the testing set (Fig. 4C, D).

The stability of the nomogram was validated using the calibration curves in the training set (Fig. 4E, F) and validation set (Fig. 4G, H). Compared with the calibration curves of ALL-model (Fig. 4E, G), the calibration plots within BIC-model (Fig. 4F, H) displayed high consistency between the actual values and estimated probability for 12-month, 36-month, and 60-month survival, respectively.

#### Comprehensive evaluation

The Brier scores in BIC model were smaller (12-month = 0.225, 36-month = 0.214, 60-month = 0.221) than the ALL model (12-month = 0.258, 36-month = 0.258, 60-month = 0.274), representing a good prediction of our nomogram.

#### Decision curve analysis

**Fig. 5:**
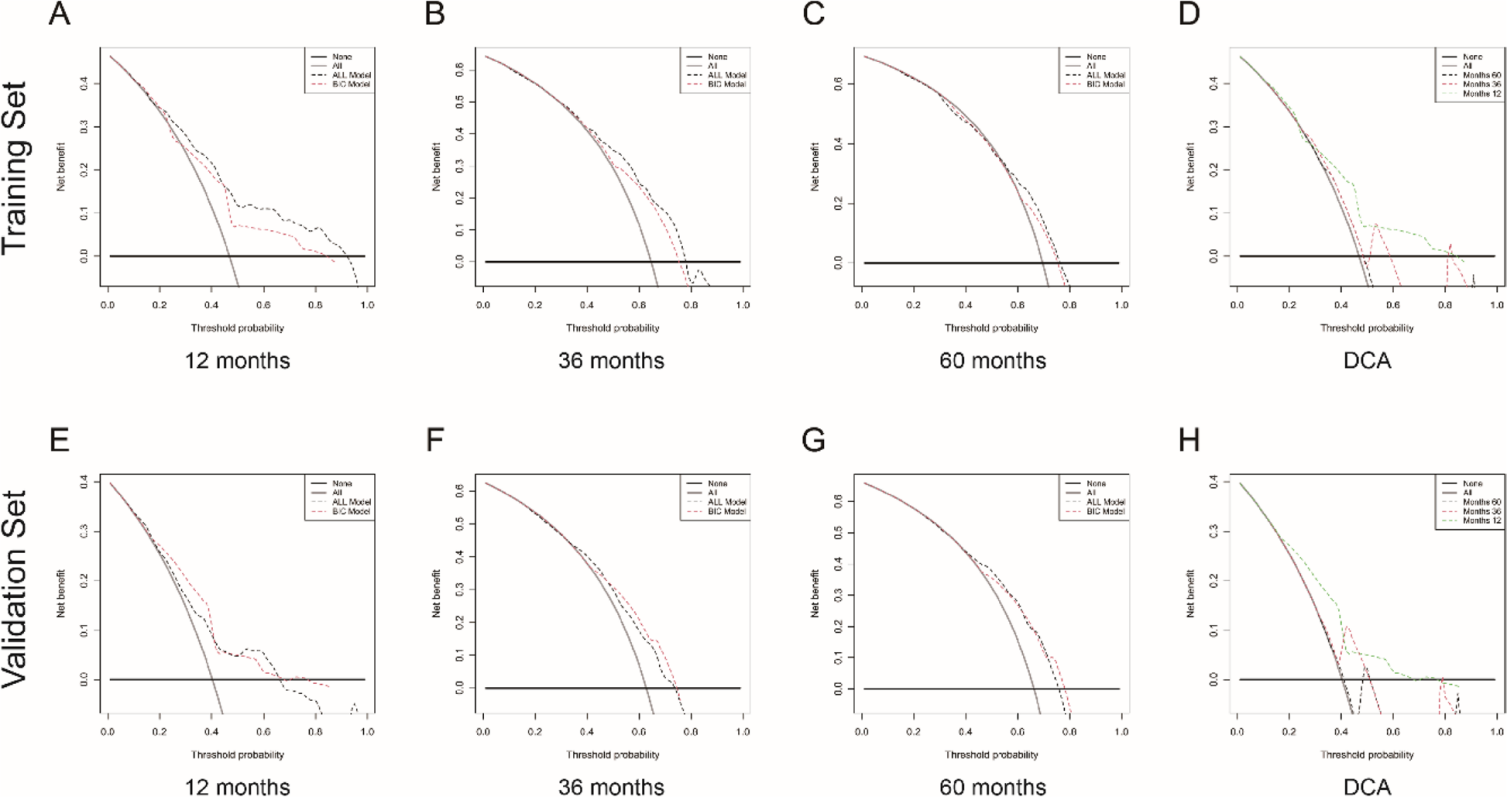
Decision curves for nomogram to predict 1-, 3-, and 5- year cancer-specific survival in the training set (**A**-**D**) and validation set (**E**–**H**). DCA, decision curve analysis; ALL-model, including all covariates; BIC-model, including selected variables

DCA presented a clinical “net benefit” for one or more predictive models compared to default interventions of treating all or no patients. Threshold probability (X-axis) is the minimum probability of which further strategy would be warranted. The “intervention for none” and “intervention for all” respectively served as negative and positive control group. When analyzing the DCA curves of 12-month, All-model performed a little better than the BIC-model in training set (Fig. 5A), whereas the opposite trend was observed in validation set (Fig. 5E). Also, there was no remarkable discrepancy regarding 36-month (Fig. 5B, F) and 60-month (Fig. 5C, G) DCA plots between two models. Overall, except for a small range of low performances of BIC-based nomogram in the training set (Fig. 5A, B, C), interventions (such as surgery) on ESRCC patients on the fundamentals of the prediction model would lead to increased clinical benefits. Furthermore, as shown in Fig. 5D and H, both two arms supported that nomogram for 1-year prediction model showed relatively more predictive values for clinical decision making.

### Subgroup analysis

**Fig. 6:**
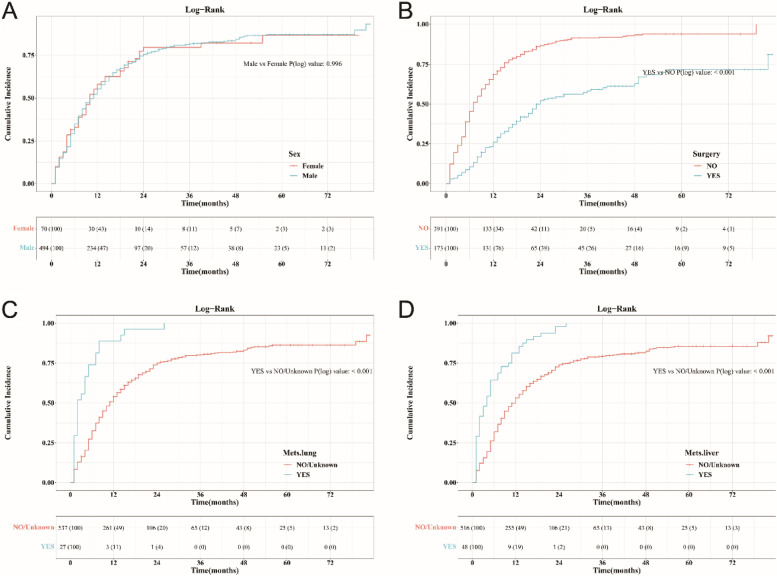
Subgroup analysis with cumulative incidence plots according to sex (**A**), receiving surgery (**B**), lung metastases (**C**), liver metastases (**D**)

We plotted the cumulative incidence curves using the data of the whole group (*N* = 564) and performed subgroup analysis on each prognostic factor. The 6-year cumulative incidence of CSS showed similar outcomes in both males and females (*P* = 0.996) (Fig. 6A). However, patients with liver metastasis, lung metastasis, or non-surgery all had a higher cumulative incidence of CSS than compared patients with statistically significant differences (*P* < 0.001), which is consistent with the findings of the multivariate SH model (Fig. 6B-D).

## Discussion

Given the competing risks event that existed, we retrospectively analyzed the SEER database and constructed a nomogram model to evaluate independent prognostic factors and predict individualized 1-year, 3-year, and 5-year ESRCC-specific survival. This nomogram model showed a favorable discriminative ability, prediction accuracy, and clinical availability in both training and validation cohorts.

The incidence of esophageal adenocarcinoma has significantly increased in the western countries [15]. Especially, ESRCC was anticipated to account for 3–3.5% of all esophageal cancers [6–8]. This rare cancer was characterized by several clinicopathological features, including age, gender, race, material status, primary site, tumor grade, tumor stage, regional lymph nodes examined, positive lymph nodes, distant metastases, treatment modalities. There are numerous studies have spotlighted on the prediction model for esophageal adenocarcinoma. For instance, one study [16] established and validated a nomogram to predict individual survival of esophageal adenocarcinoma with six variables, including tumor size, T, N, M, age, grade. Also, its nomogram presented superior risk-stratifying capability than AJCC TNM staging system.

However, a majority of them performed traditional methods to analyze the survival data, such as Kaplan–Meier curve and Cox regression model, both of which are only suitable for single time-to-event outcome. Regarding time-to-event analysis, multiple reasons can lead to death, especially for patients with a long survival time. Notably, the occurrence of one kind of death might hinder the observation of other sorts of deaths [17]. For example, one study suggested that non-lung-cancer-specific death would confound the prediction of lung-cancer-specific death. Such a bias would increase along with age [18, 19]. Therefore, the risk of interest events might be overestimate by Kaplan–Meier method and the hazard ratio would be mistakenly evaluated by Cox regression model [20]. The competing risks caused by non-ESRCC might be ignored and censored. As reported, [21] if the percentage of competing event exceeded 10%, the Kaplan–Meier curve and Cox regression model would lead to severe result biases. Consequently, introducing CSS with competing risk model when predicting the prognosis of ESRCC patients is particularly essential.

To address above issues, competing risk model, a proportional sub-distribution hazards (SH) regression model, has been frequently conducted in survival analysis. But few researches focused on ESRCC-specific survival. To fulfill the research gaps, we estimated 5-year cumulative incidence of ESRCC in the SEER database from 2010 to 2015 and constructed a well-calibrated prognostic nomogram to predict 1-year, 3-year, and 5-year CSS. Consisting with prior studies of ESRCC, predictive parameters included the gender, lung metastases, liver metastases, and receiving surgery.

In the training cohort, gender made statistical difference in ESRCC-specific mortality. Male patients tended to have a poorer CSS than female patients (HR = 2.309, 95% CI: 1.259–4.236, *P* = 0.007). But the validation cohort showed there was no association between gender and prognosis in ESRCC patients (HR = 1.641, 95% CI: 0.893–3.017, *P* = 0.11). The selection bias might explain this paradoxical finding. Female patients were significantly less than male patients in our study, consistent with previous findings indicating a higher incidence in males [22–26]. Sex-specific variants and androgen/estrogen balance associated with esophageal adenocarcinoma might improve the understanding of the reason for male predominance [27, 28]. Therefore, selection bias cannot be completely avoided owing to the different levels of disease exposure. With a limited enrollment of female patients, the median survival time is easily affected by individual difference. Overall, on the one hand, our nomogram model combined gender with other parameters showed a good discrimination and accurate prediction, supporting gender as a predictor for CSS. On the other hand, our finding indicated a poorer prognosis in men but conflicted with some studies manifesting no sex difference in prognosis observed for esophageal adenocarcinoma [28–30]. Prospective studies are urgently required to further clarify the sex difference in prognosis.

Liver (coefficient = 0.588) and lung (coefficient = 0.94) distant metastases were two predominant predictors of this competing risk nomogram, both of which acted as independent risk variables for CSS. It is because distant metastases tend to indicate high malignancy and strong invasion ability of tumor, which is connected with limited treatment options, decreased survival, and worse prognosis. A real-world study [31] reported postoperative pathologic stage might be an independent factor associated with distant metastases (*P* = 0.004, HR = 0.15, 95% CI: 0.041–0.552). Particularly, T0-T2 stage had a lower risk of metastases (*P* = 0.011, HR = 0.119, 95% CI: 0.023–0.610). Consistently, in our multivariate SH model, T3 (*P* < 0.001, HR = 4.336, 95% CI: 1.883–9.987) and T4 (*P* = 0.004, HR = 3.173, 95% CI: 1.435–7.017) stage showed a higher risk of cancer-specific mortality in the validation set. But in the training set, the wide 95% CI and P-value were unsatisfied. It is possible that T stage functions as a risk factor for distant metastasis but contributes less to ESRCC-specific survival.

Surgery (coefficient = -0.654) is the only protective factor determined in the final competing risk nomogram model. Our study suggested ESRCC patients without surgery tend to have a shorter CSS and worse prognosis than those receiving resection. Both multivariate SH analysis and cumulative incidence curves supported this finding. As known, [32] surgery is the primary option for esophageal tumor, even though only 34.6% of patients with esophageal adenocarcinoma received complete resection. Surgical methods include esophagectomy only and esophagectomy plus other excisions. A prior study supported the OS (HR = 0.366, *P* < 0.001) and CSS (HR = 0.36, *P* < 0.001) of esophageal adenocarcinoma patients who underwent surgery were significantly longer than those without surgery, which is exactly consistent with our prediction model [32]. However, a retrospective analysis [33] put forward an interesting finding. Despite receiving modern induction therapy and surgery, the five-year OS of patients with ESRCC was only 31.3%, considerably less than historically reported for esophageal adenocarcinoma. We could infer that esophagectomy is an independent protective factor for ESRCC-specific survival, although it brings fewer clinical benefits to ESRCC patients than those without the SRC signature. Notably, either chemotherapy or radiotherapy showed limited prognostic values affecting CSS in our study. Due to the lack of enough evidence, the current standard care for ESRCC is still debatable [34]. Whether advanced ESRCC patients should firstly undergo surgery resection or multimodality therapy is still unclear. However, our study provided necessary evidence that surgery could significantly improve CSS for operable ESRCC patients. It highlighted the need to increase exposure to esophagectomy during early clinical decision-making. Some patients might be unresectable during planned esophagectomy or progressed during induction therapy [33], so surgical resection should be performed as early as possible after initial diagnosis. Novel biomarkers are urgently required to predict the efficacy of esophagectomy. Further clinical evidence-based data are required to compare the benefits of chemoradiation plus surgery versus surgery alone in prospective studies.

Overall, signet-ring-cell histology is a rare subtype of esophageal adenocarcinoma, which is known to have less well response to induction therapy and have decreased OS compared to patients with non-SRC histology [9]. With the increasing incidence of ESRCC, especially for western countries, more attention should be paid to construct prognosis prediction model. Since ESRCC are commonly associated with many complications, there are a sum of possibilities leading to biased results. Therefore, a competing risk model is of particular significance. To our best knowledge, this is the first study building the competing risk nomogram for prognosis prediction of patients with ESRCC. The performance of this nomogram appears to have effective discrimination ability, adequate model calibration, and a satisfying clinical net benefit. Our study provides several significant implications for clinical practice. Firstly, based on this large population-based study, it is necessary to increase exposure to resection in the early clinical decision-making, which may bring more benefits to ESRCC patients compared to other treatment strategies. Secondly, our nomogram model is an efficient approach to predict 1-, 3-, and 5-year CSS for patients with ESRCC. All variables could be easily obtained from clinical work. By calculating the score of each feature, this model could assist clinicians to make an accurate, comprehensive, and quick prognosis judgement and develop individualized therapy for each ESRCC patient. Thirdly, this competing risk model is an economic tool to avoid medical overuse, consisting of overdiagnosis and overtreatment [35–37]. For unresectable ESRCC patients accompanying with more than one risk factor, overtreatment may place patients at risk of unnecessary adverse events. It is therefore clinicians could adopt palliative symptomatic treatment, aiming to alleviate patients’ pain and release economic burdens.

Our study has several outstanding advantages. First, our study is based on the large sample size provided by SEER database, ensuring the nomogram model robust enough. Second, cumulative incidence curve and multivariate SH model were employed to precisely estimate CSS by controlling the competing events. Third, we conducted decision curve analysis to reflect net benefit of the nomogram model, which is usually ignored in other similar reports. Meanwhile, the study has some limitations that deserves discussion. First, some recognized prognostic factors (such as genetic mutations) were not available in the SEER database. We failed to involve these parameters in our competing risk analysis. Second, since ESRCC is extremely rare in China, we lack enough data from the real world to make external validation. Third, most patients in our study were white and came from American. The universality of our competing risk model might be restricted. Finally, we only performed the internal validation for the predictive model, additional external validation is also required to guarantee its reliability and suitability.

## Conclusion

In conclusion, a competing risk nomogram for ESRCC was successfully constructed and internally validated. This model is expected to predict 1-year, 3-year, and 5-year cancer-specific survival, and help oncologists and pathologists in clinical decision making and health care management for ESRCC patients. Future studies of ESRCC connected to molecular mutations, are vital as well for developing individualized therapy and improving cancer-specific survival.

## Data Availability

The data we used in this study can be downloaded from the SEER (Surveillance, Epidemiology, and End Results) database.
